# Goldstone and Higgs modes of photons inside a cavity

**DOI:** 10.1038/srep03476

**Published:** 2013-12-11

**Authors:** Yu Yi-Xiang, Jinwu Ye, Wu-Ming Liu

**Affiliations:** 1 Beijing National Laboratory for Condensed Matter Physics, Institute of Physics, Chinese Academy of Sciences, Beijing 100190, China; 2 Department of Physics and Astronomy, Mississippi State University, P. O. Box 5167, Mississippi State, MS 39762, USA; 3 Beijing Key Laboratory for Terahertz Spectroscopy and Imaging, Key Laboratory of Terahertz Optoelectronics, Ministry of Education, Department of Physics, Capital Normal University, Beijing, 100048 China

## Abstract

Goldstone and Higgs modes have been detected in various condensed matter, cold atom and particle physics experiments. Here, we demonstrate that the two modes can also be observed in optical systems with only a few (artificial) atoms inside a cavity. We establish this connection by studying the *U*(1)/*Z*_2_ Dicke model where *N* qubits (atoms) coupled to a single photon mode. We determine the Goldstone and Higgs modes inside the super-radiant phase and their corresponding spectral weights by performing both 1/*J* = 2/*N* expansion and exact diagonalization (ED) study at a finite *N*. We find nearly perfect agreements between the results achieved by the two approaches when *N* gets down even to *N* = 2. The quantum finite size effects at a few qubits make the two modes quite robust against an effectively small counterrotating wave term. We present a few schemes to reduce the critical coupling strength, so the two modes can be observed in several current available experimental systems by just conventional optical measurements.

It was well known that a broken global continuous symmetry in quantum phases^1^,^2^ leads to two associated collective modes: the massless Goldstone mode and a massive Anderson-Higgs amplitude mode^1^,^3^,^4^ (for topological ordered phases, see^5^,^6^). The Goldstone modes have been detected in a quantum anti-ferromagnet^7^, a superfluid^8^,^9^,^10^,^11^ and also in cold atom systems^12^,^13^,^14^,^15^,^16^,^17^. However, the massive Higgs mode is much more difficult to detect in experiments. Even so, the Higgs amplitude mode was detected by Raman scattering in superconductors^18^,^19^,^20^,^21^,^22^ and by in-elastic neutron scattering in a quantum anti-ferromagnet^7^,^23^ near its quantum phase transition to a valence bond solid^24^. Unfortunately, due to the Galilean invariance, the phase mode and amplitude mode are conjugate variables, the conjugate pair only leads to a Goldstone mode. There is no Higgs mode inside a superfluid^8^,^9^,^10^,^11^,^19^,^20^,^21^,^22^. There was a report that the Higgs amplitude mode was detected in a three dimensional superfluid of strongly interacting bosons in an optical lattice by Bragg spectroscopy^25^. Most recently, the Higgs amplitude mode and its decay rate were detected in cold atoms loaded in two dimensional optical lattice near the superfluid to Mott transition by slightly modulating the lattice depth within a linear response regime^26^. Notably, the experiment detected discrete natures of the Higgs modes in a trapped system. In a relativistic quantum field theory, it is the well known Higgs mechanism^3^,^4^ which generates various mass spectrum of elementary particles. Although the various elementary particles have been discovered with the predicted masses, the original massive Higgs particle stays elusive until it was finally discovered with its mass ~ 125 *GeV* and width ~ 6 *MeV* in the recent LHC experiments^27^,^28^.

In this report, we will present the first study of the Goldstone and Higgs modes of photons inside a cavity. The conventional route is to look at how “more is different” emerges, namely, study how various macroscopic quantum phenomena emerge as the number of particles gets “more and more”^1^. Here, we will take a dual point of view: study how the emergent phenomena evolve as the number of particles becomes “less and less”. This dual approach becomes especially important in view of recent experiments of cold atoms inside an optical cavity^29^,^30^,^31^,^32^ or superconducting qubits^33^,^34^ or quantum dots^35^,^36^,^37^ inside a microcavity involving only finite to even small number of particles (Fig. 1(a)). We demonstrate this dual approach by studying the *U*(1) Dicke (Tavis-Cummings) model Eq. (1) where *N* cold atoms or qubits coupled to a single photon mode inside a cavity (Fig. 1(a)). It was known that in the thermodynamic limit^38^,^39^,^40^,^41^,^42^,^43^,^44^, when the atom-photon coupling *g* is sufficiently large, the system undergoes a quantum phase transition from a normal phase to a emergent superradiant phase which breaks the global *U*(1) symmetry. We perform both 1/*J* = 2/*N* expansion and exact diagonalization (ED) study on how the Goldstone mode and Higgs amplitude mode inside the superradiant phase evolves as the *N* decreases to a few. We find that even for a few number of *N*, the system's energy levels in the super-radiant phase display a Landau-level like structure with the inter-Landau energy scale setting by the Higgs energy *E_H_* and the intra-Landau energy scale setting by the Goldstone energy *E_G_*. In both the photon and photon number correlation functions, we evaluate the low frequency Goldstone mode *E_G_*, the high frequency Higgs mode *E_H_* and their corresponding spectral weights *C_G_* and *C_H_*. The Higgs mode is a sharp mode protected by the *U*(1) symmetry at any finite *N*. We find nearly perfect agreements between the results achieved from the 1/*J* calculations with those from the ED studies in all these physical quantities even when *N* gets down even to *N* = 2. We also study the effects of the counter rotating wave (CRW) term by the 1/*J* expansion and find that the finite size effects for a few qubits *N* ~ 2–5 dominate those of the CRW terms if *g*′/*g* < 1/3. We discuss several schemes to reduce the critical coupling, so the two modes can be observed in several experimental systems by conventional optical detection methods such as the florescence spectrum measurement^45^ on Eq. 5 and the HanburyBrown-Twiss (HBT) type of measurement^46^ on Eq. 6 respectively.

## Results

### Reducing the *U*(1)/*Z*_2_ to the *J* − *U*(1)/*Z*_2_ Dicke model

In the *U*(1)/*Z*_2_ Dicke model^47^, a single mode of photons couple to *N* two level atoms with same coupling constants 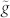 and 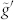. The two level atoms can be expressed in terms of 3 Pauli matrices *σ_α_*, *α* = 1, 2, 3. The *U*(1)/*Z*_2_ Dicke model can be written as: 
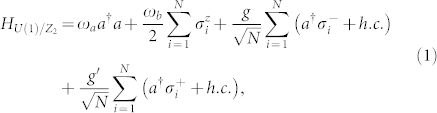
where the *ω_a_*, *ω_b_* are the cavity photon frequency and the energy difference of the two atomic levels respectively, the 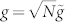 is the collective photon-atom coupling (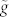 is the individual photon-atom coupling). The 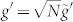 is the counter-rotating wave term. It was demonstrated in^48^,^49^ that in the thermal or cold atom experiments^29^,^30^, the strengths of *g* and *g*′ can be tuned separately by using circularly polarized pump beams in a ring cavity. In the qubit^33^,^34^ or quantum dot^35^,^36^,^37^ experiments, the CRW terms and RW terms have the same strength at the bare level, however, the CRW term is usually much smaller than the RW term at the effective level as is the case in the experiment^33^. This is because the former violates the energy conservation, while the latter respects the energy conservation, while the latter respects the energy conservation. However, when the coupling strength gets close to the the transition frequency, the CRW term becomes comparable to the RW term as is the case in the experiment in^34^. In any case, the Hamiltonian Eq. (1) with independent *g* and *g*′ is the most general Hamiltonian describing various experimental systems in various coupling regimes under the two atomic levels and a single photon mode approximation.

One can introduce the total “spin” of the *N* two level atoms 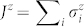, 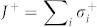, 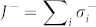, When all the *N* atoms are in the ground state, then *J* = *N*/2, *J_z_* = −*N*/2, because the total spin 
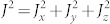
 is a conserved quantity, by confining the Hilbert space only to *J* = *N*/2, then one reduces the Hilbert space from 2*^N^* to 2*J* + 1 = *N* + 1. One can call the resulting model as the *J* − *U*(1)/*Z*_2_ Dicke model.

One main advantage of this reduction is that one can study the *J* − *U*(1)/*Z*_2_ model by using Holstein-Primakoff (HP) representation of the angular momentum operator *J_z_* = *b*^†^*b* − *J*, 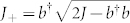, 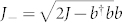, therefore treat photon and atom on the same footings. This advantage will enable us to bring out many new and important results hard to retrieve from the 1/*N* expansion in^43^. Very fortunately, this reduction will not change the most important physics of the original *U*(1)/*Z*_2_ model Eq. (1). As argued in the Methods section, except the *U*(1)/*Z*_2_ Dicke model contains some additional energy levels, both models share the same other physical quantities to be studied in this article.

If *g*′ = 0, the Hamiltonian Eq. (1) has the *U*(1) symmetry *a* → *ae^iθ^*, *σ*^−^ → *σ*^−^*e^iθ^*. The CRW *g*′ term breaks the *U*(1) to the *Z*_2_ symmetry *a* → −*a*, *σ*^−^ → −*σ*^−^. If *g*′ = *g*, it become the *Z*_2_ Dicke model studied in^44^. In this article, we focus on the *U*(1) Dicke model, but will also consider the effects of the small counter-rotating wave term *g*′ < *g* in the experimental detection section and the Methods section. The *g*′ = *g* and the *g*′ ~ *g* cases will be studied in^44^. The *U*(1) Dicke model was solved in the thermodynamic limit *N* = ∞ by various methods^38^,^39^,^40^,^41^,^42^,^43^,^44^. In the normal phase 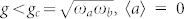, 〈*a*〉 = 0, the *U*(1) symmetry is respected. In the super-radiant phase *g* > *g_c_*, 〈*a*〉 ≠ 0, the *U*(1) symmetry is spontaneously broken.

### Goldstone and Higgs modes in the super-radiant phase by 1/J expansion

In the super-radiant phase *g* > *g_c_* and also not too close to the quantum critical point (QCP) (if too close, then 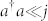, 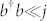, a direct 1/*j* expansion is needed and will be performed elsewhere), it is convenient to write both the photon and atom in the polar coordinates 
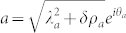
, 
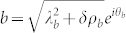
 where 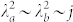. When performing the controlled 1/*J* expansion, we keep the terms to the order of ~ *j*, ~ 1 and ~ 1/*j*, but ignore orders of 1/*j*^2^. We first minimize the ground state energy at the order *j*, we found the saddle point values of *λ_a_* and *λ_b_*: 
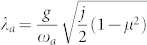
, 
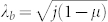
 where 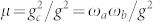. It holds only in the superradiant phase *g* > *g_c_*.

Observe that (1) in the superradiant phase *g* > *g_c_*, 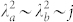, (2) it is convenient to get to the ± modes: *θ*_±_ = (*θ_a_* ± *θ_b_*)/2, *δρ*_±_ = *δρ*_a_ ± *δρ_b_*, 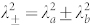. (3) paying a special attention to the crucial Berry phase term in the *θ*_+_ sector, (4) after shifting *θ*_−_ → *θ*_−_ + *π*/2, then one can get the effective action up to the order of 1/*j*: 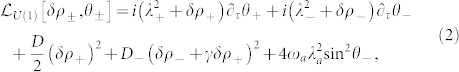
where the first line are the crucial Berry term in the *θ*_+_ and *θ*_−_ respectively, 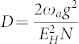 is the phase diffusion constant, 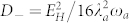 with 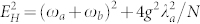. The 
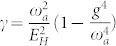
 is the coupling between the + and − sector. Under the *U*(1) transformation *θ_a/b_* → *θ_a/b_* + *χ*, *θ*_+_ → *θ*_+_ + *χ*, *θ*_−_ → *θ*_−_, so the *θ*_−_ is neutral under the *U*(1) transformation. There is a mass term for *θ*_−_, but no mass term for *θ*_+_. The conjugate pair (*θ*_+_, *δρ*_+_) leads to the Goldstone mode *E_G_* as shown in Eq. (5). While the conjugate pair (*θ*_−_, *δρ*_−_) leads to the Higgs mode *E_H_* as shown in Eq. (6) (See also the Methods section).

Defining the Berry phase in the + sector as 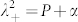 where 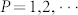 is the closest integer to the 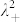, so −1/2 < *α* < 1/2. In fact, *P* = *a*^†^*a* + *b*^†^*b* is just the conserved total excitations number. Redefine 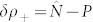, then one can write the corresponding Hamiltonian of Eq. (2) as: 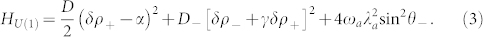
Because the *θ*_−_ is very massive, after pinning *θ*_−_ around *θ*_−_ ~ 0, one can approximate sin^2^ 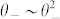, so the total wavefunction is 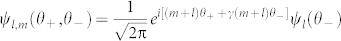 where the 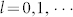 are the Landau level indices, the 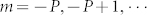 are the magnetic indices at a given sector *P*, 0 < *θ*_+_ < 2*π*, −∞ < *θ*_−_ < ∞ and the *ψ_l_*(*θ*_−_) is just the *l*-th the wavefunction of a harmonic oscillator.

The corresponding eigen-energy is 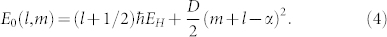
The ground state energy is at *l* = 0, *m* = 0. One can see that the energy spectrum Eq. (4) has a Landau-level structure: the Landau level energy scale is given by the Higgs energy *E_H_* ~ 1, the intra-Landau level is set up by the Goldstone energy scale *E_G_* ~ 1/*j*. In the large *j* limit, there is a wide separation of the two energy scales 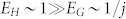. When the excitation number *P* reaches the order of *N*, then the intra-Landau levels with |*m*| ≥ *P* will start to overlap with the inter-Landau levels. These analytical results explain precisely the ED energy level structures shown in Fig. 2 for the resonant case *ω_a_* = *ω_b_*.

Away from the QCP, one can write down the 1/*J* expansion of the photon operator: 
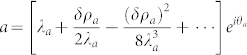
. At a finite *N*, due to the restoration of the *U*(1) symmetry by the phase diffusion in the *θ*_+_ sector, any *U*(1) non-invariant correlation functions vanish 〈*a*〉 = 0, 
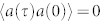
 So we need only focus on the *U*(1) invariant correlation functions. By using both canonical quantization and path integral approaches, we find the single photon correlation function: 
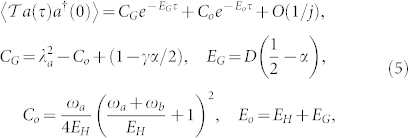
where 
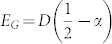
 is the Goldstone mode with the corresponding spectral weight *C_G_*, while *E_o_* = *E_H_* + *E_G_* is the optical mode with the corresponding spectral weight *C_o_*. All these quantities can be directly measured by the florescence spectrum measurement^45^.

The *E_G_*, *C_G_* and *E_o_*, *C_o_* are compared with the ED results in Fig. 3 and Fig. 4 respectively. One can see that except at the first few 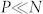 steps, the ED in *E_o_* match the analytical relation *E_o_* = *E_H_* + *E_G_* in Eq. (5) well. The discrepancy at the first few steps is not surprising, as said previously, if too close to the QCP, a direct 1/*j* expansion is needed and will be performed elsewhere. However, the agreement between the analytical and ED results in *C_o_* holds in all couplings even near the QCP.

One can also compute the photon number correlation function: 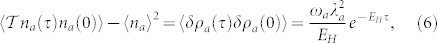
where 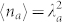. The Higgs energy *E_H_* and the corresponding spectral weight 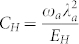 are compared with the ED results in Fig. 5. Note that the sharpness of the Higgs mode is protected by the conservation of *δρ*_+_ in Eq. (3). Both *C_H_* and *E_H_* can be directly measured by the HanburyBrown-Twiss (HBT) type of measurement on two photon correlation functions^46^.

**Figure 1:**
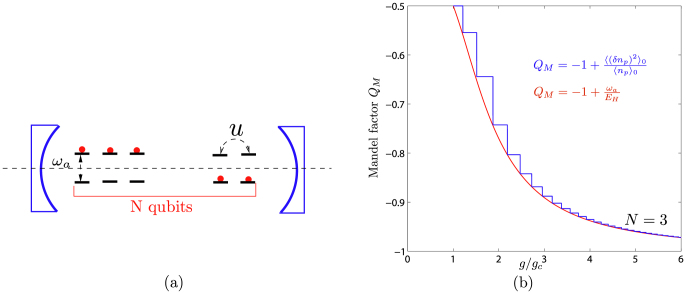
(a) *N* (artificial) atoms are placed on anti-nodes of a cavity. *u* is the repulsive qubit-qubit interaction which can be tuned to reduce the critical coupling *g_c_*. (b) The analytical Mandel factor *Q_M_* (red) against the ED result (blue) at *N* = 3. It is a number squeezed state inside the superradiant phase.

From the Eq. (6), one can see that 
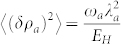
, so one can find the Mandel *Q* factor: 
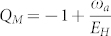
 which was compared with the ED result in the Fig. 1(b). For *ω_a_* = ω*_b_*, one can see −1 < *Q_M_* < −1/2. So it is always in a number squeezed state. As *g* → ∞ limit, *Q_M_* → −1, so it approaches a photon Fock state. It is known that number squeezed states could be very important in quantum information processing and also in high-resolution and high sensitivity measurements. Very similarly, one can evaluate the atom correlation functions.

### Effects of the CRW term and experimental detections of the Goldstone and Higgs modes

The effects of the CRW terms on system's energy Eq. (4), photon correlation function Eq. (5) and the number correlation function Eq. (6) are examined in the Methods section. Their effects were found to be much smaller than those of the finite size for a few qubits *N* ~ 2 − 5 if *g*′/*g* < 1/3. Recent experiments^29^,^30^ reached the *Z*_2_ super-radiant regime^44^ with the help of a transverse pumping. In this transverse pumping scheme, the CRW terms in Eq. (1) are as important as the RW ones *g*′ = *g*, so only the *Z*_2_ super-radiant phase can be realized. However, it was demonstrated in^48^,^49^ that the strengths of *g*′ and *g* can be tuned independently by using circularly polarized pump beams in a ring cavity. So we expect that *g*′/*g* < 1/3 can be achieved in this transverse pumping scheme, then the system can be tuned to the *U*(1) superradiant regime. It is also promising to reach the *Z*_2_ superradiant regime “simultaneously” (namely without any transverse pumping) with artificial atoms such as superconducting qubits inside micro-wave circuit cavity^33^,^34^ and quantum dots inside a semi-conductor nano-cavity engraved in a photonic crystal in Fig. 1(a)^35^,^36^,^37^. Indeed, very recently, by enhancing the inductive coupling of a flux qubit to a transmission line resonator, a remarkable ultra-strong coupling with individual 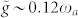 was realized in a circuit QED system^33^,^34^. In this simultaneous scheme, due to the violation of the energy conservation, the CRW term is usually much smaller than the RW one *g*′ < *g*, but gets stronger as the coupling gets stronger. The effects of the CRW terms on system's energy Eq. (4), photon correlation function Eq. (5) and the number correlation function Eq. (6) are examined in the Methods section. Their effects were found to be much smaller than those of the finite size for a few qubits *N* ~ 2 − 5 if *g*′/*g* < 1/3. In real experiments of superconducting qubits or quantum dots inside a cavity in Fig. 1(a), there are always the potential scattering term *λ_z_J_z_a*^†^*a*/*j* between the cavity photons and the qubits and the qubit-qubit interaction term 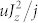. The critical coupling *g_c_* is shifted to: 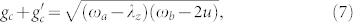
which indicates that the two repulsive interaction terms decrease the critical *g_c_* well below the bare critical frequency 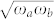. The qubit-qubit interactions can be tuned inductively or capacitively. This fact could be used to put the system into the regime where the CRW term satisfies *g*′/*g* < 1/3, so the *U*(1) super-radiant phase can be realized in the possible future experiments using both atoms inside a optical cavity or qubits inside a microwave circuit QED in Fig. 1(a).

There are also other promising experimental systems to realize the *U*(1) super-radiant phase. Most recently, the giant dipole moments of intersubband transitions in quantum wells have pushed the system into the ultrastrong light-matter coupling regime in semiconductor heterostructures^50^,^51^. Very recent experiments^52^ achieved very strong coupling between an ensemble of *s* = 1/2 spins and photons in electronic spin ensembles coupled to superconducting cavities. The strong coupling regimes are also realized in ion Coulomb crystals in an optical cavity^53^.

## Discussion

Quantum mechanics describes the motion of a single or a few particles^54^,^55^,^56^,^57^,^58^. Condensed matter physics studies various emergent quantum phenomena of macroscopic number of interacting particles. Ultracold atom systems and optical cavity systems can provide unprecedented experimental systems to study quantum phenomena ranging from a few particles to a million number of interacting particles. Due to the tremendous tunability of all the parameters in these systems, they can be tuned to scale up from the isolated quantum mechanics systems to macroscopic condensed matter systems. In this article, we show that the many body theory developed to study the emergent phenomena of condensed matter systems can also be a very powerful tool to study the physical phenomena from millions of particles down even to a few particles. Especially, we study how the emergent Goldstone and Higgs modes evolve as the number of particles gets less and less, even down only a few particles in quantum optical systems. The discrete natures of both modes shown in Fig. 3 and Fig. 5 at a finite *N* are due to the Berry phase effects −1/2 < *α* < 1/2. Both modes are well defined sharp quasi-particle excitations with no damping. Especially, the sharpness of the Higgs mode is the *U*(1) symmetry protected at any finite *N*. We also found that the finite size effects at a few qubits making the *U*(1) super-radiant phase quite robust against the counter rotating wave (CRW) term. Both modes can be detected even with a few qubits or ions inside a QED microwave cavity by conventional optical measurements^45^,^46^,^59^. Considering it is difficult to scale up these systems to large number of qubits at the present technologies, this feature becomes experimentally appealing. Our theoretical works should provide a solid foundation for various ongoing and upcoming systems with a small number of particles to observe the novel phenomena due to strong light-matter interactions explored in this report.

## Methods

### Exact diagonalization (ED) study

For simplicity, in the following, we limit our ED study only to the resonant case *ω_a_* = *ω_b_*. We assume *P* ≤ *N*. The *P* > *N* case can be similarly addressed by changing *P* + 1 to *N* + 1. The ground state in the given *P* Hilbert space is: 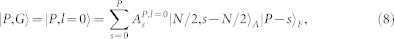
where the coefficients 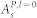 can be determined by the ED. From Eq. (8), one can evaluate the Mandel *Q* factor *Q_M_* = −1 + 〈(*δn_p_*)^2^〉/〈*n_p_*〉 which was compared with the analytical result in Fig. 1(b).

The *l*-th eigen-state in the *P* + 1 sector with the eigen-energy 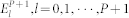 is: 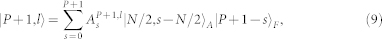
where the coefficients 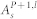 can be determined by the ED.

In the Lehmann representation, we can evaluate the photon-photon correlation function: 
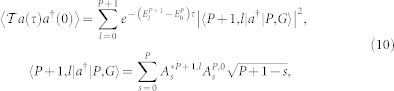
where 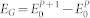 is the Goldstone mode with the corresponding spectral weight *C_G_* = |〈*P* + 1, *l* = 0|*a*^†^|*P*, *l* = 0〉|^2^, while 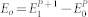 is the optical mode with the corresponding spectral weight *C_o_* = |〈*P* + 1, *l* = 1|*a*^†^|*P*, *l* = 0〉|^2^ and so on. In fact, there are *P* + 2 lines, we just focus on the two lowest energy excitations *l* = 0, 1.

Very similarly, one can evaluate the photon number correlation function: 
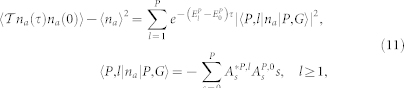
where 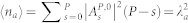 and the Higgs mode 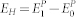 with the spectral weight *C_H_* = |〈*P*, *l* = 1|*n_a_*|*P*, *G*〉|^2^. Very similarly, one can evaluate the atom correlation functions.

### Relations between *J* − *U*(1)/*Z*_2_ Dicke model and the *U*(1)/*Z*_2_ model

**Figure 2:**
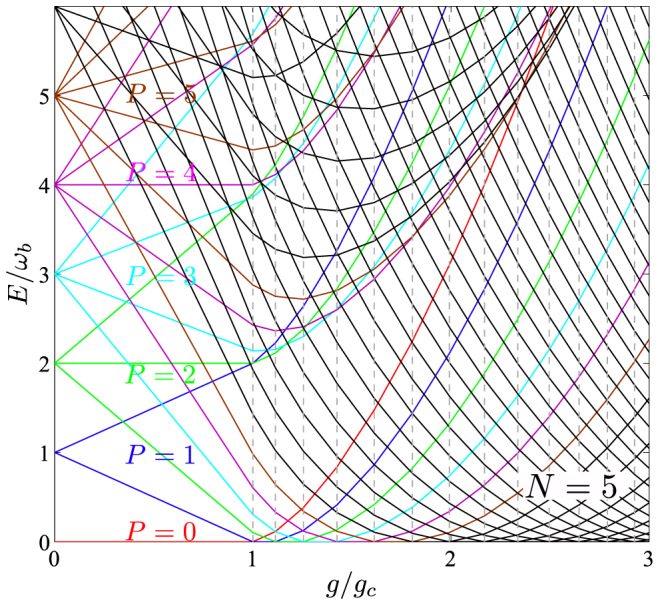
The ED results (See the Methods section) of the energy levels *E* measured by subtracting the ground-state energy versus *g*/*g_c_* at resonance *ω_a_* = *ω_b_* with *N* = 5 atoms. Different colors of the energy curves correspond to several smallest numbers of total excitations number *P* = *a*^†^*a* + *b*^†^*b*. The dashed vertical lines correspond to the critical values of *g* where the number of total excitations *P* in the ground state increases by one.

The energy levels in the lowest Landau level (LLL) shown in Fig. 2 are identical in *U*(1) and *J* − *U*(1) models. This is because the ground state must be a totally symmetric state. In fact, every ground state in a given *P* = *a*^†^*a* + *b*^†^*b* sector must be a totally symmetric state. It is the crossings of all these ground states at different *P* sectors which lead to all the energy levels in the LLL shown in the Fig. 2. This explains why the diffusion constant *D* achieved by 1/*J* expansion in this article is identical to that achieved by the 1/*N* expansion in Ref. 43. Because both photon and total spin operators are also totally symmetric in the atom operators, then all the energy levels coupled to the ground state by the photon and total spin operators are also totally symmetric, so this also explains why we achieved the same single photon or atom correlation functions in the reduced Hilbert space in the *J* − *U*(1) Dicke model by 1/*J* expansion as those in the whole Hilbert space by the 1/*N* expansion. However, compared to the reduced Hilbert space in the *J* − *U*(1) Dicke model, there are many extra energy levels in the whole Hilbert space in the *U*(1) Dicke model, but they are not coupled to the ground state by the single photon or atom operators. Similar arguments apply to the more general *U*(1)/*Z*_2_ model with the CRW term in Eq. (1) and the *J* − *U*(1)/*Z*_2_ model.

### Comparisons with the Higgs mode and pseudo-Goldstone mode in one gap and two gaps superconductors

It is constructive to compare the Goldstone and Higgs mode of the atom-photo system studied in this report with those in (charge neutral) superconductors (so one can ignore the Anderson-Higgs mechanism for the sake of explaining physical concepts). In a one gap superconductor, as explicitly demonstrated in the last reference in Ref. 19,20,21,22, when integrating out the fermions, the amplitude and phase of the paring order parameter ψ = Δ*e^iθ^* emerges as two *independent* degree of freedoms, instead of being conjugate to each other. Its phase fluctuation in *θ* leads to the Goldstone mode, while its amplitude fluctuation in Δ leads to the Higgs mode.

Now we consider the collective modes in a two gap superconductor such as *M_g_B*_2_ which has a *σ* band and a *π* band. Therefore it has two order parameters 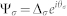 and 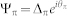. There are also fermionic degree of freedoms: *σ* electrons and *π* electrons. If ignoring the interband scattering *V_σ_*_,*π*_, the Hamiltonian has two independent *U*(1) symmetries: *U*(1)*_σ_* × *U*(1)*_π_*, the systems is just two copies of single band superconductor. So there are two independent Goldstone modes.

*θ_σ_*, *θ_π_* and also two independent Higgs modes Δ*_σ_*, Δ*_π_* for the two bands respectively. Now when considering the interband scattering term *V_σ_*_,*π*_, the symmetry of the Hamiltonian reduces from *U*(1)*_σ_* × *U*(1)*_π_* to [*U*(1)*_σ_* × *U*(1)*_π_*]*_D_* where the *D* means the simultaneous rotation of the two order parameter phases. Then the two Goldstone modes couple to each other and split into one gapless Goldstone mode *θ*_+_ = *θ_σ_* + *θ_π_* plus a gapped pseudo-Goldstone mode *θ*_−_ = *θ_σ_* − *θ_π_*. The pseudo-Goldstone mode *θ*_−_ is just the relative phase mode between the two order parameters whose gap is proportional to the strength of the interband scattering *V_σ_*_,*π*_. The two Higgs modes Δ*_σ_*, Δ*_π_* will also couple to each other and split into two new Higgs modes. In all, the two gaps superconductor has one gapless Goldstone mode and 3 gapped modes: one pseudo-Goldstone mode and two Higgs modes.

A pseudo-Goldstone mode is always associated with an explicit symmetry breaking of a Hamiltonian, its gap is proportional to the strength of the explicit symmetry breaking.

In contrast, a Higgs mode is the magnitude fluctuations of an order parameter. It is always associated with a spontaneous symmetry breaking in a ground state. The final physical meaning of a relative phase mode depends on the physical degree of freedoms of a system and its original relation to the order parameters of the system. As shown below Eq. (2) in the main text, the conjugate pair (*δρ_−_*, *θ*_−_) fluctuation leads directly to the photon amplitude fluctuation mode, namely, the Higgs mode in Eq. (6).

To some extent, the photon-atom system studied here is similar to one gap superconductor discussed in^19^,^20^,^21^,^22^ with the photon corresponding to the pairing order parameter, while the atoms corresponding to the fermions. When integrating out the atomic degree freedoms, the amplitude and phase of the photon order parameter emerges as two *independent* degree of freedoms, instead of being conjugate to each other. Its phase fluctuation leads to the Goldstone mode, while its amplitude fluctuation leads to the Higgs mode. This fact was demonstrated by the 1/*N* expansion in^43^ and also by Eq. (5) and Eq. (6) of this report by 1/*J* expansion. As shown in the Methods section, a small counter-rotating wave *g*′ term in Eq. (1) break sthe *U*(1) symmetry to a *Z*_2_ symmetry, then the Goldstone mode at *N* = ∞ will become a pseudo-Goldstone mode whose gap is proportional to the strength of the counter-rotating wave term.

### The effects of the counter-rotating wave term at *N* = ∞ and at a finite *N*

Now we consider the effects of the counter-rotating wave (CRW) terms in Eq. (1). Following the same procedures in the main text, we find that 
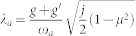
, 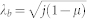 where *μ* = *ω_a_ω_b_*/(*g* + *g*′)^2^, so the QCP is shifted to 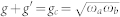. The Hamiltonian to the order of 1/*j* is: 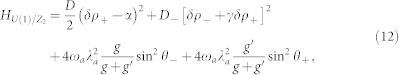
where 
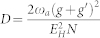
 is the phase diffusion constant, 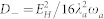 with 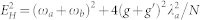. The 
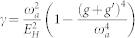
 is the coupling between the + and − sector.

Eq. (12) can be rewritten as 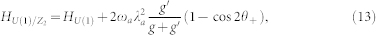
where *H_U_*_(1)_ takes the same form as Eq. (3) with the parameters corrected by *g*′. The last CRW term breaks the *U*(1) symmetry to *Z*_2_ symmetry *θ_a/b_* → *θ_a/b_* + *π*, *θ*_+_ → *θ*_+_ + π, *θ*_−_ → *θ*_−_, so the *θ*_−_ is neutral under the *Z*_2_ transformation. In the thermodynamic limit *N* = ∞, it leads to a small mass term for *θ*_+_, so the Goldstone mode at *N* = ∞ becomes a pseudo-Goldstone mode with a small gap 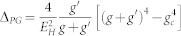. Obviously, this gap vanishes at the QCP *g* + *g*′ = *g_c_*. In the following, we discuss its effects at a finite *N*.

If we ignore the CRW term, all the results achieved in the main text on the systems's energies Eq. (4), the photon correlation function Eq. (4) and the photon number correlation function Eq. (6) remain intact after making the corresponding changes in the parameters. Then for small *g*′/*g*, at a finite *N*, we can can treat the CRW term by the perturbation theory. Here, we only list the main results. Obviously, the high energy Higgs mode is in-sensitive to this CRW term, so we only need to focus on its effect on the low energy Goldstone mode. Then the sole dimensionless small parameter is 
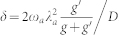
. (1) For the Berry phase *α* ≠ 0, non-degenerate perturbation leads to the correction to the system's eigen-energy Eq. (4) at the second order ~ *δ*^2^. Note that although at *α* = −1/2, the energy is doubly degenerate with (*δρ*_+_ = *m*, *δρ*_+_ = −*m* − 1), but *m* and −*m* − 1 carry opposite parities, so they will not be mixed by the CRW term. So the nondegenerate perturbation theory is valid. For the Berry phase *α* = 0, because the two degenerate states (*m*, −*m*), *m* > 0 carry the same parity, one need to use the degenerate perturbation theory to treat their splitting. The pair (*m*, −*m*) will split only at the *m*−the order degenerate perturbation, so the splitting Δ*E* ~ *δ^m^*. (2) The normal photon correlation function Eq. (5) receives a correction ~ *δ*^2^ in both energy and spectral weight. Most importantly, there appears also an anomalous photon correlation function 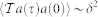. So the detection of a small anomalous photon correlation function by phase sensitive homodyne experiments^45^,^46^,^59^. could be used to determine the strength of the CRW term.

**Figure 3:**
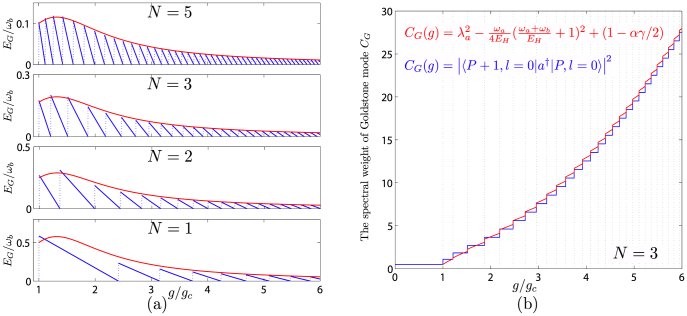
(a) The analytical Goldstone mode at *α* = −1/2, 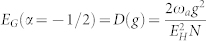 (red line) are contrasted with the ED result 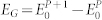 (blue lines) at *N* = 5, 3, 2, 1 respectively. It is remarkable that the analytical result can even map out broad peaks at small *P* in the ED results very precisely. (b) The analytical spectral weight (red) of the Goldstone mode *C_G_* against the ED result (blue) at *N* = 3.

**Figure 4:**
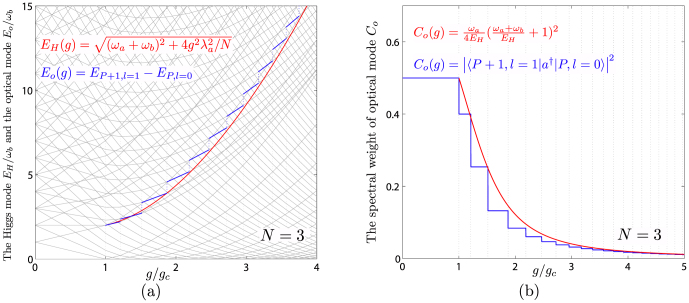
(a) The analytical relation *E_o_* = *E_H_* + *E_G_* (*E_H_* in red line) is satisfied by the ED optical mode 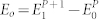 (blue lines) at *N* = 3 except at the first few steps. (b) The analytical spectral weight (red) of the optical mode *C_o_* against the ED result (blue) at *N* = 3.

**Figure 5:**
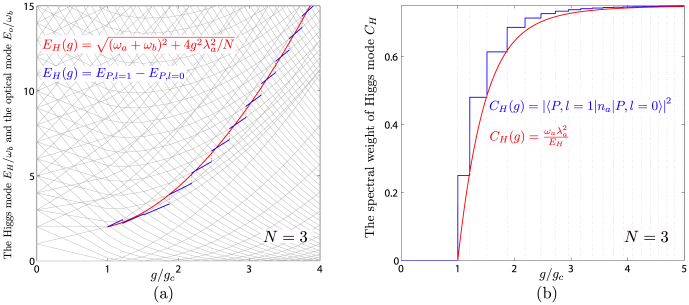
(a) The analytical Higgs energy *E_H_* (red) against the ED result 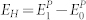 (blue) at *N* = 3. (b) The analytical spectral spectral weight *C_H_* (red) for the Higgs mode against the ED result (blue) at *N* = 3.

One can see that the corrections to all the physical quantities are at the second order ~ *δ*^2^ or higher. From the *N* = 2 qubits in the Fig. 3(a), one can see that *D* ~ *ω_a_*/4, 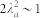 near the QCP, then when *g*′/*g* < 1/3, the corrections due to the CRW term is suppressed compared to the finite size effects. Physically, at *N* = ∞, any CRW term will transform the gapless Goldstone mode into a pseudo-Goldstone mode whose gap is proportional to the strength of the CRW term. In contrast, at a finite *N*, the quantum finite size effects already opened a gap to the Goldstone mode which is of the phase diffusion constant *D* ~ 1/*N*. This gap make the Goldstone in a finite system *N* = 2 – 5 quite robust against the CRW term if *g*′/*g* < 1/3.

## Author Contributions

J.Y. designed this work and wrote the manuscript. J.Y. and Y.Y.-X. performed the theoretical and numerical research. W.-M.L. commented and revised the manuscript. All authors reviewed the manuscript.

## References

[b1] Anderson P. W. Basic Notions of Condensed Matter Physics (Benjamin-Cummings, London, 1984).

[b2] Sachdev S. Quantum Phase Transitions (Cambridge University Press, Cambridge, U.K., 1999).

[b3] Englert F. & Brout R. Broken Symmetry and the Mass of Gauge Vector Mesons. Phys. Rev. Lett. 13, 508–509 (1964).

[b4] Guralnik G. S., Hagen C. R. & Kibble T. W. B. Global Conservation Laws and Massless Particles. Phys. Rev. Lett. 13, 585–587 (1964).

[b5] Hasan M. Z. & Kane C. L. Colloquium: Topological insulators. Rev. Mod. Phys. 82, 3045–3067 (2010).

[b6] Qi X. L. & Zhang S. C. Topological insulators and superconductors. Rev. Mod. Phys. 83, 1057–1110 (2011).

[b7] Chubukov A. V., Sachdev S. & Ye J. Theory of two-dimensional quantum Heisenberg anti-ferromagnets with a nearly critical ground state. Phys. Rev. B 49, 11919–11961 (1994). 10.1103/physrevb.49.11919 10010065

[b8] Ye J. & Jiang L. Quantum Phase Transitions in Bilayer Quantum Hall Systems at a Total Filling Factor *ν_T_* = 1. Phys. Rev. Lett. 98, 236802 (2007). 17677927 10.1103/PhysRevLett.98.236802

[b9] Ye J. Quantum Phases of Excitons and Their Detections in Electron-Hole Semiconductor Bilayer Systems. J. Low Temp. Phys. 158(5), 882–900 (2010).

[b10] Ye J. Elementary Excitations, Spectral Weights and Experimental Signatures of a Supersolid and a Fulde-Ferrell-Larkin-Ovchinnikov State. J. Low Temp. Phys. 160(3), 71–111 (2010).

[b11] Ye J., Zhang K. Y., Li Y., Chen Y. & Zhang W. P. Optical Bragg, atom Bragg and cavity QED detections of quantum phases and excitation spectra of ultracold atoms in bipartite and frustrated optical lattices. Ann. Phys. 328, 103–138 (2013).

[b12] Kozuma M. *et al.* Coherent Splitting of Bose-Einstein Condensed Atoms with Optically Induced Bragg Diffraction. Phys. Rev. Lett. 82, 871–875 (1999).

[b13] Ernst P. T. *et al.* Probing superfluids in optical lattices by momentum-resolved Bragg spectroscopy. Nature Phys. 6, 56–61 (2010).

[b14] Stamper-Kurn D. M. *et al.* Excitation of Phonons in a Bose-Einstein Condensate by Light Scattering. Phys. Rev. Lett. 83, 2876–2879 (1999).

[b15] Steinhauer J., Ozeri R., Katz N. & Davidson N. Excitation Spectrum of a Bose-Einstein Condensate. Phys. Rev. Lett. 88, 120407 (2002). 11909437 10.1103/PhysRevLett.88.120407

[b16] Papp S. B. *et al.* Bragg Spectroscopy of a Strongly Interacting ^85^Rb Bose-Einstein Condensate. Phys. Rev. Lett. 101, 135301 (2008). 18851457 10.1103/PhysRevLett.101.135301

[b17] Stenger J. *et al.* Bragg Spectroscopy of a Bose-Einstein Condensate. Phys. Rev. Lett. 82, 4569–4573 (1999).

[b18] Sooryakumar R. & Klein M. V. Raman scattering by superconducting-gap excitations and their coupling to charge-density waves. Phys. Rev. Lett. 45, 660–662 (1980).

[b19] Littlewood P. B. & Varma C. M. Gauge-invariant theory of the dynamical interaction of charge density waves and superconductivity. Phys. Rev. Lett. 47, 811–814 (1981).

[b20] Littlewood P. B. & Varma C. M. Amplitude collective modes in superconductors and their coupling to charge-density waves. Phys. Rev. B 26, 4883–4893 (1982).

[b21] Varma C. M. Higgs Boson in superconductors. J. Low Temp. Phys. 126, 901–909 (2002).

[b22] Aitchison I. J. R., Ao P., Thouless D. J. & Zhu X. M. Effective Lagrangians for BCS superconductors at *T* = 0. Phys. Rev. B 51, 6531–6535 (1995). 10.1103/physrevb.51.6531 9977188

[b23] Podolsky D. & Sachdev S. Spectral functions of the Higgs mode near two-dimensional quantum critical points. Phys. Rev. B 86, 054508 (2012).

[b24] Rüegg C. *et al.* Quantum magnets under pressure: controlling elementary excitations in TlCuCl_3_. Phys. Rev. Lett. 100, 205701 (2008). 18518554 10.1103/PhysRevLett.100.205701

[b25] Bissbort U. *et al.* Detecting the amplitude mode of strongly interacting lattice bosons by Bragg scattering. Phys. Rev. Lett. 106, 205303 (2011). 21668240 10.1103/PhysRevLett.106.205303

[b26] Endres M. *et al.* The Higgs amplitude mode at the two-dimensional superfluid/Mott insulator transition. Nature 487, 454–458 (2012). 22837000 10.1038/nature11255

[b27] ATLAS Collaboration, Observation of a new particle in the search for the Standard Model Higgs boson with the ATLAS detector at the LHC. Phys. Lett. B 716, 1–29 (2012).

[b28] CMS Collaboration, Observation of a new boson at a mass of 125 GeV with the CMS experiment at the LHC. Phys. Lett. B 716, 30–61 (2012).

[b29] Black A. T., Chan H. W. & Vuletic V. Observation of Collective Friction Forces due to Spatial Self-Organization of Atoms: From Rayleigh to Bragg Scattering. Phys. Rev. Lett. 91, 203001 (2003). 14683358 10.1103/PhysRevLett.91.203001

[b30] Baumann K. *et al.* Dicke quantum phase transition with a superfluid gas in an optical cavity. Nature 464, 1301–1306 (2010). 20428162 10.1038/nature09009

[b31] Bakr W. S. *et al.* Probing the Superfluid-to-Mott Insulator Transition at the Single-Atom Level. Science 30, 547–550 (2010). 20558666 10.1126/science.1192368

[b32] Serwane F. *et al.* Deterministic Preparation of a Tunable Few-Fermion System. Science 15, 336–338 (2011). 21493855 10.1126/science.1201351

[b33] Wallraff A. *et al.* Strong coupling of a single photon to superconducting qubit using circuit quantum elctrodynamics. Nature 431, 162–167 (2004). 15356625 10.1038/nature02851

[b34] Niemczyk T. *et al.* Circuit quantum electrodynamics in the ultrastrong-coupling regime. Nature Phys. 6, 772–776 (2010).

[b35] Reithmaiser J. P. *et al.* Strong coupling in a single quantum dot-semi-conductor micro-cavity system. Nature 432, 197–200 (2004). 15538362 10.1038/nature02969

[b36] Yoshie T. *et al.* Vacuum Rabi splitting with a single quantum dot in a photonic crystal nanocavity. Nature 432, 200–203 (2004). 15538363 10.1038/nature03119

[b37] Hennessy K. *et al.* Quantum nature of a strongly coupled single quantum dot-cavity system. Nature 445, 896–899 (2007). 17259971 10.1038/nature05586

[b38] Hepp K. & Lieb E. H. On the Superradiant Phase Transition for Molecules in a Quantized Radiation Field: The Dicke Maser Model. Ann. Phys. (N. Y.) 76, 360–404 (1973).

[b39] Wang Y. K. & Hioe F. T. Phase Transition in the Dicke Model of Superradiance. Phys. Rev. A 7, 831–836 (1973).

[b40] Popov V. N. & Fedotov S. A. The functional integration method and diagram technique for spin systems. Soviet Physics JETP 67, 535–541 (1988).

[b41] Popov V. N. & Yarunin V. S. Collective Effects in Quantum Statistics of Radiation and Matter (Kluwer Academic, Dordrecht, 1988).

[b42] Buzek V., Orszag M. & Rosko M. Instability and entanglement of the ground state of the Dicke model. Phys. Rev. Lett. 94, 163601 (2005). 15904225 10.1103/PhysRevLett.94.163601

[b43] Ye J. & Zhang C. L. Super-radiance, Photon condensation and its phase diffusion. Phys. Rev. A 84, 023840 (2011).

[b44] Emary C. & Brandes T. Chaos and the quantum phase transition in the Dicke model. Phys. Rev. E 67, 066203 (2003). 10.1103/PhysRevE.67.066203 16241322

[b45] Ye J., Shi T. & Jiang L. Angle-Resolved Photoluminescence Spectrum of the Exciton Condensate in Electron-Hole Semiconductor Bilayers. Phys. Rev. Lett. 103, 177401 (2009). 19905781 10.1103/PhysRevLett.103.177401

[b46] Shi T., Jiang L. & Ye J. Phase sensitive two-mode squeezing and photon correlations from exciton superfluid in semiconductor electron-hole bilayer systems. Phys. Rev. B 81, 235402 (2010).

[b47] Dicke R. H. Coherence in Spontaneous Radiation Processes. Phys. Rev. 93, 99–110 (1954).

[b48] Dimer F., Estienne B., Parkins A. S. & Carmichael H. J. Proposed realization of the Dicke-model quantum phase transition in an optical cavity QED system. Phys. Rev. A 75, 013804 (2007).

[b49] Bhaseen M. J., Mayoh J., Simons B. D. & Keeling J. Dynamics of nonequilibrium Dicke models. Phys. Rev. A 85, 013817 (2012).

[b50] Günter G. *et al.* Sub-cycle switch-on of ultrastrong light-matter interaction. Nature 458, 178–181 (2009). 19279631 10.1038/nature07838

[b51] Anappara A. A. *et al.* Signatures of the ultrastrong light-matter coupling regime. Phys. Rev. B 79, 201303 (2009).

[b52] Schuster D. I. *et al.* High-Cooperativity Coupling of Electron-Spin Ensembles to Superconducting Cavities. Phys. Rev. Lett. 105, 140501 (2010). 21230817 10.1103/PhysRevLett.105.140501

[b53] Herskind P. F. *et al.* Realization of collective strong coupling with ion Coulomb crystals in an optical cavity. Nature Phys. 5, 494–498 (2009).

[b54] Bergquist J. C., Hulet R. G., Itano W. M. & Wineland D. J. Observation of Quantum Jumps in a Single Atom. Phys. Rev. Lett. 57, 1699–1702 (1986). 10033522 10.1103/PhysRevLett.57.1699

[b55] Monroe C., Meekhof D. M., King B. E., Itano W. M. & Wineland D. J. Demonstration of a Fundamental Quantum Logic Gate. Phys. Rev. Lett. 75, 4714–4717 (1995). 10059979 10.1103/PhysRevLett.75.4714

[b56] Brune M. *et al.* Quantum Rabi Oscillation: A Direct Test of Field Quantization in a Cavity. Phys. Rev. Lett. 76, 1800–1803 (1996). 10060524 10.1103/PhysRevLett.76.1800

[b57] Brune M. *et al.* Observing the Progressive Decoherence of the Meter in a Quantum Measurement. Phys. Rev. Lett. 77, 4887–4890 (1996). 10062660 10.1103/PhysRevLett.77.4887

[b58] Brune M., Haroche S., Raimond J. M., Davidovich L. & Zagury N. Manipulation of photons in a cavity by dispersive atom-field coupling: Quantum-nondemolition measurements and generation of “Schrödinger cat” states. Phys. Rev. A 45, 5193–5214 (1992). 9907607 10.1103/physreva.45.5193

[b59] Ye J., Sun F., Yu Y.-X. & Liu W.-M. Exciton correlations and input-output relations in non-equilibrium exciton superfluids. Ann. Phys. 329, 51–72 (2013).

